# The Initiation Factors eIF2, eIF2A, eIF2D, eIF4A, and eIF4G Are Not Involved in Translation Driven by Hepatitis C Virus IRES in Human Cells

**DOI:** 10.3389/fmicb.2018.00207

**Published:** 2018-02-13

**Authors:** Esther González-Almela, Hugh Williams, Miguel A. Sanz, Luis Carrasco

**Affiliations:** Centro de Biología Molecular Severo Ochoa (CSIC-UAM), Universidad Autónoma de Madrid, Madrid, Spain

**Keywords:** regulation of protein synthesis, initiation factor of translation, inhibitors of eIF2, regulation of viral translation, eIF2 phosphorylation

## Abstract

Animal viruses have evolved a variety of strategies to ensure the efficient translation of their mRNAs. One such strategy is the use of internal ribosome entry site (IRES) elements, which circumvent the requirement for some eukaryotic initiation factors (eIFs). Much effort has been directed to unravel the precise mechanism of translation initiation by hepatitis C virus (HCV) mRNA. In the present study, we examined the involvement of several eIFs in HCV IRES-driven translation in human cells in a comparative analysis with mRNAs bearing the encephalomyocarditis virus or the Cricket paralysis virus IRES element. Consistent with previous findings, several inhibitors of eIF2 activity, including sodium arsenite, thapsigargin, tunicamycin, and salubrinal, had no inhibitory effect on the translation of an mRNA bearing the HCV IRES, and all induced the phosphorylation of eIF2α. In addition, hippuristanol and pateamine A, two known inhibitors of eIF4A, failed to block HCV IRES-directed translation. To test the release of nuclear proteins to the cytoplasm and to analyze the formation of stress granules, the location of the nuclear protein TIA1 was tested by immunocytochemistry. Both arsenite and pateamine A could efficiently induce the formation of stress granules containing TIA1 and eIF4G, whereas eIF3 and eIF2 failed to localize to these cytoplasmic bodies. The finding of eIF4A and eIF4G in stress granules suggests that they do not participate in mRNA translation. Human HAP1 cells depleted for eIF2A, eIF2D, or both factors, were able to synthesize luciferase from an mRNA bearing the HCV IRES even when eIF2α was phosphorylated. Overall, these results demonstrate that neither eIF2A nor eIF2D does not participate in the translation directed by HCV IRES. We conclude that eIF2, eIF4A, eIF2A, and eIF2D do not participate in the initiation of translation of HCV mRNA.

## Introduction

Hepatitis C virus (HCV) is responsible for the vast majority of chronic viral hepatitis and induces hepatocarcinoma in humans (Hajarizadeh et al., 2013; Khullar and Firpi, 2015). HCV belongs to the *Flaviviridae* family and contains a 9.6 kb single-stranded RNA of positive polarity as its genome. Its genomic RNA is the only known viral mRNA and bears a single open reading frame (ORF) encoding for a large polyprotein, which after proteolytic processing renders the mature viral proteins that participate in genome replication and in the assembly of new virus particles (Paul et al., 2014). Translation of HCV mRNA is promoted and regulated by an internal ribosome entry site (IRES) element that mediates the internal initiation of translation by supporting the interaction of components that participate in protein synthesis (Hellen and Pestova, 1999; Khawaja et al., 2015). Results from *in vitro* experiments initially suggested that the first step in the initiation of this viral mRNA involved the recruitment of initiation factors eIF3, eIF2, eIF5, GTP, initiator tRNA_i_^Met^ and a 40S ribosomal subunit by HCV IRES, yielding a 43S preinitiation complex (Pestova et al., 1998; Otto and Puglisi, 2004). Precise attachment of this complex at the initiation AUG codon forms a 48S complex in a process that does not involve eIF4F or the scanning of the 5′-UTR. The HCV mRNA has the ability to interact directly with the 40S ribosomal subunit, recruiting then eIF3 and the ternary complex. In this process, two modules of the IRES region, domains II and III, are necessary for the interaction with the small ribosomal subunit and eIF3 (Lukavsky, 2009; Khawaja et al., 2015; Yamamoto et al., 2015). Also, interaction of the HCV mRNA with preinitiation complexes bearing eIFs can take place, in a process that displaces eIF2, but requires eIF1A and eIF3 (Jaafar et al., 2016). Subsequently, the 60S ribosomal subunit interacts with this complex in a process mediated by eIF5B, which induces the release of eIF3 and leads to the formation of the 80S initiation complex, ready to start the elongation process. This mechanism of internal initiation is in sharp contrast to the canonical initiation of cellular capped mRNAs. In this latter instance, the initiation of protein synthesis begins with the recognition of the cap structure by the eIF4F complex, which contains eIF4E, the cap recognition protein, eIF4G, a scaffolding protein, and eIF4A, which exhibits helicase activity in an ATP-dependent manner (Topisirovic et al., 2011). Once eIF4F is bound to the cap structure at the 5′ end of cellular mRNAs, the small 40S ribosomal subunit bearing eIF3 and the ternary complex eIF2-Met-tRNA_i_^Met^-GTP interact with the mRNA. In addition, other factors such as eIF1, eIF1A, and eIF5 bind to the small ribosomal subunit forming the 48S complex. Then, this complex scans the 5′-UTR until the initiator AUG codon is encountered (Sonenberg and Hinnebusch, 2009; Hinnebusch et al., 2016). Joining of the 60S ribosomal subunit is promoted by eIF5B concomitant with the release of the eIFs in a GTP-dependent manner. Aside from the requirement of only a few eIFs for the translation of HCV mRNA, a number of IRES *trans*-acting factors, which modulate HCV mRNA translation have been reported. These factors include NSAP1, La protein, hnRNP L and D, Gemin5, LSm1-7, IMP-1 and PCBP2; although their exact mechanism of action in the translation of this viral mRNA remains largely unknown (Niepmann, 2013).

The participation of eIF2 in the initiation of HCV mRNA translation is controversial. In principle, two different mechanisms can be followed: translation of HCV mRNA takes place with eIF2 when this factor is active under normal conditions; yet, IRES-driven translation occurs after inactivation of this factor by phosphorylation under stress conditions. Initial studies using reconstituted translation systems indicated that eIF2 was necessary for the translation of this viral mRNA *in vitro* (Pestova et al., 1998; Hellen and Pestova, 1999). Moreover, analyses using mRNAs bearing HCV IRES in cell free systems revealed the presence of eIF2 in the initiation complexes (Otto and Puglisi, 2004). However, the interaction of this viral IRES with preinitiation complexes displaces eIF2 from them (Jaafar et al., 2016). That said, a novel class of inhibitors of the formation of the ternary complex had no effect on HCV IRES-driven translation, whereas these compounds potently interfered with canonical protein synthesis (Robert et al., 2006). In addition, stress conditions that promote the phosphorylation of eIF2α and block cellular protein synthesis did not compromise HCV mRNA translation (Terenin et al., 2008; Kim et al., 2011; Dabo and Meurs, 2012; Jaafar et al., 2016). In light of this, several candidates have been put forward to replace eIF2 for protein synthesis promoted by HCV IRES under stress conditions. For example, eIF5B can substitute for eIF2 *in vitro* in the delivery of Met-tRNA_i_^Met^ to small ribosomal subunits directed by HCV mRNA (Terenin et al., 2008). Under these conditions, the initiation of protein synthesis by HCV mRNA only requires two initiation factors: eIF3 and eIF5B. Another proposal suggested that eIF2D can substitute for eIF2 when this factor is inactivated (Dmitriev et al., 2010; Skabkin et al., 2010). However, the possibility that eIF5B or eIF2D participate in the initiation of HCV mRNA in intact cells under stress conditions was not analyzed, and only *in vitro* observations were reported. The involvement of eIF2A for the translation of HCV mRNA in place of eIF2 in intact cells and in cell free systems, has also been proposed (Kim et al., 2011). Accordingly, Huh-7 cells depleted for eIF2A cannot translate luciferase driven by the HCV IRES in a bicistronic mRNA when eIF2α is phosphorylated. In contrast to these findings, recent results have shown that depletion of eIF2A or eIF2D or both factors in Huh-7 cells have no effect for the translation of HCV mRNA (Jaafar et al., 2016). Under stress conditions, only eIF1A, eIF5B, and eIF3 should be necessary to direct the synthesis of proteins by this viral mRNA. It has been speculated that under conditions in which eIF2 is non-functional, the initiator Met-tRNA_i_^Met^ can bind directly to the ribosome following a mechanism that does not require the ternary complex, but is directed by HCV IRES (Jaafar et al., 2016).

Although eIF2A and eIF2D can form a complex with Met-tRNA_i_^Met^ and deliver it to 40S or 80S ribosomes, their involvement in translation remains obscure. Indeed, early results demonstrated that eIF2A can interact with Met-tRNA_i_^Met^ and deliver it to the ribosome (Merrick and Anderson, 1975). However, this binding was much less efficient than that observed using genuine eIF2 on artificial templates and eIF2A was unable to promote the binding of Met-tRNA_i_^Met^ to globin mRNA (Adams et al., 1975). Moreover, a complex between Met-tRNA_i_^Met^ and eIF2D is formed in a GTP-independent fashion, which can interact with the 40S ribosomal subunit to deliver the initiator to the P site of the ribosome (Dmitriev et al., 2010). eIF2D could displace deacylated tRNA and mRNA from recycled 40S ribosomal subunits, and was also able to interfere with the formation of the 48S initiation complex promoted by eIF2 (Skabkin et al., 2010). Both eIF2A and eIF2D are 65 kDa proteins. Deletion of the yeast ortholog of eIF2A or eIF2D has no effect on cell viability (Zoll et al., 2002; Dmitriev et al., 2010). Consistent with these observations, human cells depleted for the genes encoding these two initiation factors are also viable and global protein synthesis is unaffected (Sanz et al., 2017). Results from mammalian cells have suggested that eIF2A is involved in the translation of specialized cellular mRNAs that initiate translation with non-AUG codons (Liang et al., 2014; Starck et al., 2016). Elegant studies have recently implicated eIF2A in cancer progression because it is involved in the initiation of translation of upstream ORFs (Sendoel et al., 2017). However, mice deleted for the eIF2A gene are completely normal, supporting the concept that eIF2A is not necessary for the translation of both normal and specialized cellular mRNAs (Golovko et al., 2016).

Here, we examined the involvement of several eIFs in HCV IRES-driven translation in human cells. Our findings indicate that knock out human cells for eIF2A, eIF2D, or both, are not only viable, but also synthesize proteins in a manner similar to that of wild-type cells. In addition, by investigating the potential involvement of these two proteins for the translation of HCV mRNA, we demonstrate that these factors are not required for translation of this viral mRNA, even when eIF2α is phosphorylated.

## Materials and Methods

### Cell Lines

Huh-7 cells are a well differentiated hepatocyte-derived cellular carcinoma cell line established by Nakabayashi et al. (1982). Cells were cultured in Dulbecco’s Modified Eagle’s Medium (DMEM, Thermo Fisher Scientific, Waltham, MA, United States) supplemented with non-essential amino acids, 4 μM glutamine, 10% fetal calf serum, 50 U mL^-1^ penicillin and 50 U mL^-1^ streptomycin.

Wild-type (WT) HAP1 human near-haploid cells and knock-out HAP1 cells for eIF2A (cat# HZGHC002650c001), eIF2D (cat# HZGHC002652c005) or double knock-cells (cat# HZGHC005122c010) were purchased from Horizon Discovery Group plc (Cambridge, United Kingdom). The eIF2A knockout (KO) cell line (gi|977380191|ref|NM_032025.4|) has a 16 bp deletion in exon 4 resulting in a frameshift that generates a protein of 108 amino acids in place of the original protein of 585 amino acids. The eIF2D KO cell line (gi|56699484|ref|NM_006893.2|) has a 10 bp deletion in exon 3 resulting in a frameshift that generates a protein of 103 amino acids in place of the original protein of 584 amino acids. The double KO line has the same 16 bp deletion in exon 4 of the single eIF2A KO cell line and a 22 bp deletion in exon 3 of eIF2D that generates a protein of 99 amino acids in place of the 584 amino acid protein. Cells were cultured in Iscove’s Modified Dulbecco’s Medium (IMDM, Invitrogen, Carlsbad, CA, United States) supplemented with 10% fetal calf serum.

All cell lines were maintained at 37°C with 95% humidity and 5% CO_2_.

### Plasmids and Reporter Constructs

The pT7HCV33core-Luc vector was kindly donated by Dr. Takashi Shimoike (National Institute of Infectious Diseases, Musashimurayama, Tokyo). It contains nucleotides 1–374 of the HCV genome followed by the firefly luciferase gene and finally the 3′ UTR of HCV. The construct is transcribed from a T7 polymerase promoter that precedes these sequences (Shimoike et al., 2009). The gene segments from HCV comprise the 5′ UTR containing the IRES, followed by the first 33 nucleotides of the HCV Core protein encoding a sequence that has previously been shown to be crucial for the proper function of the IRES (Reynolds et al., 1995). HCV-Luc RNA was *in vitro* transcribed from pT7HCV33core-Luc.

The pTM1-Luc vector was derived from pTM1 (Moss et al., 1990), and was constructed as described (Sanz et al., 2010). It contains a modified form of the encephalomyocarditis virus (EMCV) IRES along with the firefly luciferase gene. EMCV-Luc RNA was *in vitro* transcribed from pTM1-Luc.

The pT7-RLuc-ΔEMCV-IGR-FLuc vector has been previously described (Redondo et al., 2011). It contains the T7 promoter followed by the *Renilla* luciferase gene and a deactivated form of the EMCV IRES; it also contains the intergenic region of cricket paralysis virus (CrPV) followed by the firefly luciferase gene. CrPV-Luc RNA was *in vitro* transcribed from pT7-RLuc-ΔEMCV-IGR-FLuc.

The β-globin construct contains the leader sequence from the human β-globin gene followed by the firefly luciferase gene. A Cap.βGlobin-Luc transcript was obtained by *in vitro* transcription using pKS-GL-FL as a template, as described by Castello et al. (2009). This plasmid was kindly provided by Dr. Matthias Hentze and Dr. Francesca Moretti (EMBL, Heidelberg, Germany).

The pFKi389LucNS3-3_dg_JFH vector, which was kindly donated by Dr. Ralph Bartenschlager (Department of Molecular Virology, University of Heidelberg, Heidelberg, Germany), was used to obtain rep HCV-Luc RNA by *in vitro* transcription. This plasmid contains the T7 promoter sequence fused to nucleotides 1–389 of the JFH-1 consensus sequence, followed by the firefly luciferase gene, the EMCV IRES, the NS3-to-NS5B coding sequence, the 3′ NTR of JFH-1, the hepatitis delta virus genomic ribozyme (dg), and the T7 terminator sequence (Schaller et al., 2007). All plasmids contain the ampicillin resistance gene for selection purposes.

### *In Vitro* Transcription

Plasmids were linearized with the appropriate restriction enzymes (pT7HCV33core-Luc: BamHI, pT7-RLuc-ΔEMCV-IGR-FLuc: BamHI, pTM1-Luc: XhoI, pKS-GL-FL: HindIII; pFKi389LucNS3-3_dg_JFH: MluI). All restriction enzymes were purchased from New England Biolabs (Ipswich, MA, United States). Linearized plasmids were used as templates for *in vitro* RNA transcription using T7 or T3 RNA polymerases (New England Biolabs, Ipswich, MA, United States), the m7G(5′)ppp(5′)G cap analog (New England Biolabs) was used for Cap.βGlobin-Luc transcription. Mixtures were incubated for 2 h at 37°C. *In vitro*-synthesized RNAs were treated with recombinant DNase I (RNase-free) (Takara Bio USA Inc., Terra Bella, CA, United States) for 30 min at 37°C. All transcripts were transfected using Lipofectamine 2000 reagent (Invitrogen, Carlsbad, CA, United States) following the manufacturer recommendations.

### Inhibitors

Pateamine A [purified as described (Bordeleau et al., 2005)] and hippuristanol (Bordeleau et al., 2006b) were kindly provided by J. Pelletier (McGill University, Montreal, QC, Canada). Sodium arsenite was obtained from Riedel-de Haën (Hanover, Germany), and thapsigargin, tunicamycin, salubrinal, and cycloheximide were purchased from Sigma–Aldrich (St. Louis, MO, United States). Inhibitors are described in **Table 1**.

**Table 1:** eIF2 inhibitors utilized in this study and the mechanisms by which they increase eIF2α phosphorylation.

Inhibitor	Mechanism of action
Sodium arsenite	Induces phosphorylation of elF2α by activating heme-regulated inhibitor kinase, a member of the elF2α-specific kinase subfamily.
Thapsigargin	Triggers the release of calcium to the cytoplasm from the endoplasmic reticulum, activating PERK which in turn phosphorylates elF2.
Tunicamycin	Inhibits protein glycosylation and leads to endoplasmic stress.
Salubrinal	Inhibits the PP1/GADD34 complex which is known to dephosphorylate elF2α.


### Luciferase Activity Assay

Cells were lysed in a buffer containing 0.5% Triton X-100, 25 mM glycylglycine pH 7.8, 1 mM dithiothreitol and complete EDTA-free protease inhibitor cocktail (Roche Molecular Systems Inc., Pleasanton, CA, United States) at the concentration indicated by the supplier. Luciferase activity was determined using the Luciferase Assay System (Promega, Madison, WI, United States) and a Sirius Luminometer (Titertek-Berthold, Pforzheim, Germany).

As a control, cycloheximide (CHX) was added to block translation, in order to determine the luciferase synthesized in the absence of compounds during the 1st hour of transfection.

### Antibodies

Goat polyclonal anti-TIA-1 (C-20) (catalog number sc-17519), rabbit polyclonal anti-eIF2α (catalog number sc-11386), mouse monoclonal anti-eIF2α (catalog number sc-133132), goat polyclonal anti-eIF3 (catalog number sc-16376), mouse monoclonal anti-eIF4A (catalog number sc-14211), goat polyclonal anti-eIF1 (catalog number sc-390122) antibodies were purchased from Santa Cruz Biotechnology (Dallas, TX, United States). Rabbit monoclonal anti-eIF1A antibody (catalog number ab172623) was purchased from Abcam (Cambridge, United Kingdom). Rabbit polyclonal anti-eIF2D antibody (catalog number 12840-1-AP) was purchased from Proteintech Group, Inc. (Rosemont, IL, United States). Rabbit polyclonal anti-eIF2A antibody (catalog number A301-949A-M) was purchased from Bethyl Laboratories Inc. (Montgomery, TX, United States). Rabbit polyclonal anti-phospho-eIF2α (serine 51) antibody (catalog number 9721) was purchased from Cell Signaling Technology Inc. (Danvers, MA, United States). Rabbit polyclonal antibody anti-eIF4GI has been obtained as previously described (Aldabe and Carrasco, 1995).

Anti-rabbit immunoglobulin G antibody coupled to peroxidase was purchased from Amersham (catalog number NA934V) (GE Healthcare, Chicago, IL, United States). Specific antibodies conjugated to Alexa 488 or Alexa 555 (A-21202 and A-21432, respectively) were obtained from Invitrogen (Carlsbad, CA, United States).

### Immunocytochemistry and Confocal Microscopy

Fixation, permeabilization and confocal microscopy were performed as described by Madan et al. (2008) using the LSM 710 confocal laser scanning and multiphoton microscope coupled to an inverted microscope (Axio Observer, Zeiss, Oberkochen, Germany). Bound primary antibodies were detected by secondary antibodies coupled to Alexa 488 or Alexa 555 (Molecular Probes Inc., Eugene, OR, United States). Nuclei were stained with DAPI (4′-6-diamidino-2-phenylindole). All images were collected and analyzed using Zeiss ZEN 2010 software.

### Western Blotting

Cells were collected in sample buffer, boiled for 5 min and processed by SDS-PAGE. After electrophoresis, proteins were transferred to nitrocellulose membranes. Specific rabbit polyclonal antibodies raised against phospho-eIF2α (Ser 51), total eIF2α, eIF2A, and eIF2D were used at 1:1000 dilution in TBS with 3% bovine serum albumin and 0.1% Tween 20. Anti-rabbit immunoglobulin G antibody coupled to peroxidase (Amersham GE Healthcare, Chicago, IL, United States) was used as secondary antibody at a 1:5000 dilution. Protein bands were visualized with the ECL detection system (Amersham, GE Healthcare).

### Statistical Analysis

Data analysis was performed using Excel (Microsoft, Redmond, WA, United States) and GraphPad Prism 6 (GraphPad Software Inc., La Jolla, CA, United States) softwares. Data are shown as mean with standard error. Statistical validation was done using two-way analysis of variance (ANOVA) followed by a Bonferroni *post hoc* test or one-way ANOVA with Tukey’s *post hoc* test. Statistical significance is shown as: ^∗^*p* < 0.05, ^∗∗^*p* < 0.01, ^∗∗∗^*p* < 0.001.

## Results

### HCV IRES-Driven Translation Is Refractory to Inhibitors That Induce the Phosphorylation of eIF2α in Human Hepatic Cells

We sought to investigate the behavior of HCV IRES regarding its dependence on several eIFs under appropriate physiological conditions. To accomplish this, we used the Huh-7 human hepatoma cell line. We first tested a monocistronic mRNA encoding for luciferase, bearing the HCV IRES at the 5′ end and containing the 3′-UTR of this RNA (HCV-Luc). The presence of the 3′-UTR is important because it is involved in modulating translation of HCV mRNA (Ito et al., 1998; Bai et al., 2013; Shwetha et al., 2015). To reproduce conditions similar to those found during HCV infection, we also tested a replicon of HCV (rep HCV-Luc), which contains the firefly luciferase gene, the EMCV IRES, the NS3-to-NS5B coding sequence, the 3′ NTR of JFH-1 and the hepatitis delta virus genomic ribozyme (dg) (Schaller et al., 2007). As controls, we employed an mRNA bearing the CrPV intergenic region (IGR) (CrPV-Luc), which does not require eIFs to initiate protein synthesis (Jan and Sarnow, 2002; Fernandez et al., 2014). We also employed the IRES of EMCV (EMCV-Luc), which uses eIF2 and eIF4A for translation (Welnowska et al., 2011), and a capped mRNA containing the globin 5′-leader sequence (Cap.βGlo-Luc), which follows the canonical mechanism for its translation. All these mRNAs encode luciferase as a reporter gene (Supplementary Figure 1).

**FIGURE 1:**
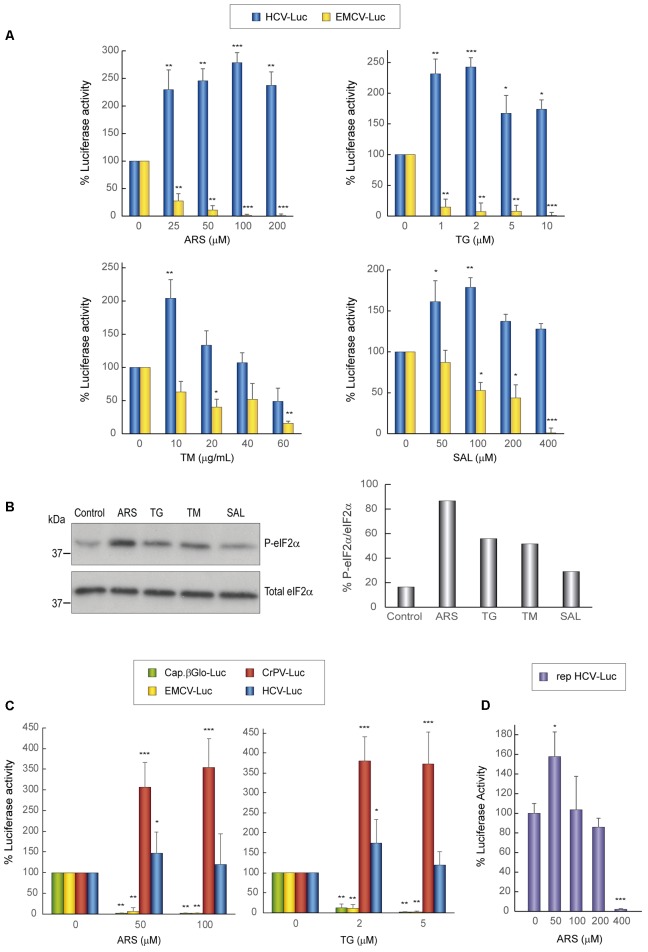
Translation from the HCV IRES in Huh-7 cells is resistant to the action of eIF2 inhibitors. **(A)** Translation from the IRES of HCV or EMCV as measured by luciferase activity in response to eIF2 inhibitor treatment in Huh-7 cells. Cells were transfected with *in vitro* synthesized HCV-Luc or EMCV-Luc mRNAs for 1 h and then incubated with either cycloheximide CHX (5 μg mL^-1^) or ARS, TG, SAL, or TM for a further 2 h. Percentage change is relative to that in the non-treated control (inhibitor concentration = 0). The readings from CHX treatments were subtracted from all as a baseline. Error bars represent the standard error of the mean, *n* = 3. **(B)** Inhibitor treatment induces eIF2 phosphorylation in Huh-7 cells. Cells were transfected with *in vitro* synthesized HCV-Luc RNA as above and then treated or not with ARS (100 μM), TG (5 μM), TM (40 μg mL^-1^), or SAL (200 μM) for 2 h. Proteins were resolved by SDS-PAGE and blots were probed with antibodies against phospho-eIF2α and total eIF2α. Shown is a representative blot from three independent experiments. The phosphorylation of eIF2α induction rate was evaluated by normalizing the raw value of P-eIF2α to that of total eIF2α as shown in the bar graph. **(C)** Huh-7 cells were transfected with different *in vitro* transcribed reporter RNAs: HCV-Luc, EMCV-Luc, CrPV-Luc or Cap.βGlobin-Luc. After 1 h of transfection, cells were treated or not with ARS or TG, or with CHX, after which luciferase activity was measured. Bars represent the relative luciferase activity with non-treated control (inhibitor concentration = 0) set as 100%. The readings from CHX treatments were subtracted from all as a baseline. Error bars represent the standard error of the mean, *n* = 3. **(A,C)** Statistical significance of the differences between treated samples compared to control was calculated with two-way ANOVA and a Bonferroni *post hoc* test, and is shown as: ^∗^*p* < 0.05, ^∗∗^*p* < 0.01, ^∗∗∗^*p* < 0.001. **(D)** Huh-7 cells were transfected with *in vitro* synthesized rep HCV-Luc mRNA. After 1 h of transfection, cells were treated with different concentrations of ARS or CHX (5 μg mL^-1^) for 2 h, after which luciferase activity was measured. Bars represent the relative luciferase activity with non-treated control (inhibitor concentration = 0) set as 100%. The readings from CHX treatments were subtracted as a baseline. Error bars represent the standard error of the mean, *n* = 3. Statistical significance of the differences between treated samples compared to control was calculated with one-way ANOVA and a Tukey’s *post hoc* test, and is shown as: ^∗^*p* < 0.05, ^∗∗∗^*p* < 0.001.

Initially, we explored the action of different concentrations of several compounds that induce the phosphorylation of eIF2α. Accordingly, Huh-7 cells were first transfected with HCV-Luc and EMCV-Luc mRNAs for 1 h and the following compunds were added to the culture medium for a further 2 h: sodium arsenite (ARS), thapsigargin (TG), tunicamycin (TM), and salubrinal (SAL). The first three compounds activate kinases that induce eIF2α phosphorylation, whereas SAL inhibits eIF2α dephosphorylation (Boyce et al., 2005; Sanz et al., 2009; Vaughn et al., 2014; Garcia-Moreno et al., 2015). As a control, cycloheximide (CHX) was added to block translation, in order to determine the luciferase synthesized in the absence of compounds during the 1st hour of transfection. The four compounds tested blocked luciferase synthesis directed by EMCV IRES in a concentration-dependent manner, albeit to different extents and with different inhibitory concentrations (**Figure 1A**). By contrast, the same compounds exerted a stimulatory effect on HCV-Luc mRNA translation. Among the compounds, ARS and TG were the most potent inhibitors/stimulators. Accordingly, 100 μM ARS almost entirely inhibited EMCV-Luc translation and stimulated HCV-Luc translation by ∼250%. A similar result was obtained with 2 μM TG. TM also stimulated luciferase production from HCV-Luc ∼200% at 10 μg mL^-1^, but the same concentration inhibited EMCV-Luc translation by only 30%. Higher concentrations of this compound further decreased the translation of EMCV-Luc, whereas the translation of HCV-Luc was partially inhibited. Perhaps this inhibition is due to the toxicity of TM at these high concentrations. A moderate inhibition of luciferase activity from EMCV-Luc was observed with SAL up to 200 μM (∼40%), and activity was completely inhibited at 400 μM. Conversely, the activity of HCV-Luc was stimulated ∼150–175% or remained unaltered in relation to the control.

To assess whether the compounds induced the phosphorylation of eIF2α, we performed western blotting on cell extracts using specific antibodies. A robust increase in eIF2α phosphorylation in Huh-7 cells was found with the four agents assayed, with the most potent being ARS (**Figure 1B**). We next tested the activity of the two most potent inhibitors, ARS and TG, on four different mRNAs: HCV-Luc, EMCV-Luc, CrPV-Luc, and Cap.βGlo-Luc. Two concentrations of the inhibitors were added 1 h after transfection of the different mRNAs, and CHX was also added as a control as before. We found that protein synthesis directed by both EMCV-Luc and Cap.βGlo-Luc was effectively blocked by both compounds (**Figure 1C**). Notably, HCV-Luc was stimulated by ARS and TG ∼150%. A comparable response was also found with CrPV-Luc mRNA, albeit with a stronger stimulation, overall indicating a similar behavior of both mRNAs in the presence of these inhibitors as regards to their dependence on eIF2. This stimulation of HCV and CrPV IRESs is probably due to the inhibition of global cellular translation by these compounds, thus reducing cellular mRNA competition.

The translation driven by rep HCV-Luc mRNA in the presence of ARS was also tested. To do this, Huh-7 cells were transfected with rep HCV-Luc mRNA for 1 h, and subsequently different concentrations of ARS were added to the culture medium for a further 2 h. The luciferase synthesis directed by rep HCV-Luc was stimulated ∼158% by treatment with 50 μM ARS (**Figure 1D**), which is in good agreement with the results obtained with HCV-Luc, suggesting that HCV does not require eIF2 for its translation under replication conditions. Curiously, ARS concentrations above 200 μM were inhibitory for rep HCV translation. This result suggests that some differences exist between the translation of the HCV-Luc mRNA and the rep HCV, which bears most of the viral coding sequences.

### Requirement of eIF4A for IRES-Driven Translation

**FIGURE 2:**
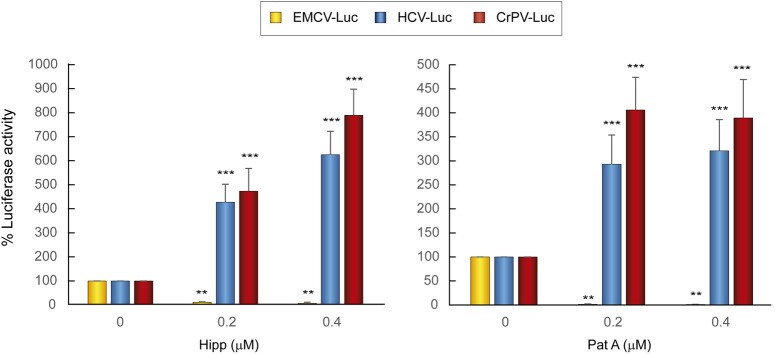
Translation from the HCV IRES in Huh-7 cells is resistant to the action of eIF4A inhibitors. Huh-7 cells were transfected with reporter RNAs (HCV-Luc, EMCV-Luc, or CrPV-Luc) for 1 h and then treated with either Hipp (0.2 and 0.4 μM) or Pat A (0.2 and 0.4 μM) for 2 h, after which luciferase activity was measured. Bars represent the relative luciferase activity with non-treated control (inhibitor concentration = 0) set as 100%. The readings from CHX treatments were subtracted from all as a baseline. Error bars represent the standard error of the mean, *n* = 3. Statistical significance of the differences between treated samples compared to control was calculated with two-way ANOVA and a Bonferroni *post hoc* test, and is shown as: ^∗∗^*p* < 0.01, ^∗∗∗^*p* < 0.001.

Two natural compounds of marine origin, hippuristanol (Hipp) and pateamine A (Pat A), have been characterized as potent blockers of eIF4A activity (Bordeleau et al., 2006a; Low et al., 2007). eIF4A is the helicase subunit of the eIF4F complex, which additionally contains eIF4E and eIF4G (Topisirovic et al., 2011). Both inhibitors have been shown to exhibit a dual inhibitory effect on some viral mRNAs (Garcia-Moreno et al., 2013; Gonzalez-Almela et al., 2015). To analyze the action of Hipp and Pat A on luciferase synthesis driven by the HCV, EMCV and CrPV IRES elements, different concentrations of these compounds were added 1 h after transfection for a further 2 h. Consistent with previous works (Garcia-Moreno et al., 2013; Gonzalez-Almela et al., 2015), both Hipp and Pat A potently blocked EMCV IRES-dependent luciferase synthesis in human hepatic cells. By contrast, both compounds had no detrimental effect on translation driven by HCV or CrPV IRESs in these cells, and in fact strongly stimulated translation (**Figure 2**). These results are consistent with the concept that eIF4F does not participate in protein synthesis directed by HCV IRES (Hellen, 2009; Niepmann, 2013).

### Cellular Localization of eIFs Treated with Sodium Arsenite or Pateamine A

It is well established that ARS and Pat A induce the formation of cytoplasmic stress granules (SGs). The molecular mechanism of this induction is different for each compound: ARS induces eIF2α phosphorylation, whereas SG formation by Pat A occurs via a mechanism independent of this process (Dang et al., 2006; Linero et al., 2011). A number of components of the translation machinery, including preinitiation complexes containing 40S ribosomal subunits, are present in SGs (Kedersha and Anderson, 2009; Anderson et al., 2015; Penas et al., 2016). It was of note that HCV-Luc mRNA was efficiently translated even in the presumed presence of SGs (**Figures 1**, **2**). Curiously, HCV infection of Huh-7 cells leads to a dynamic oscillation in the formation of SGs (Ruggieri et al., 2012; Valadao et al., 2016).

**FIGURE 3:**
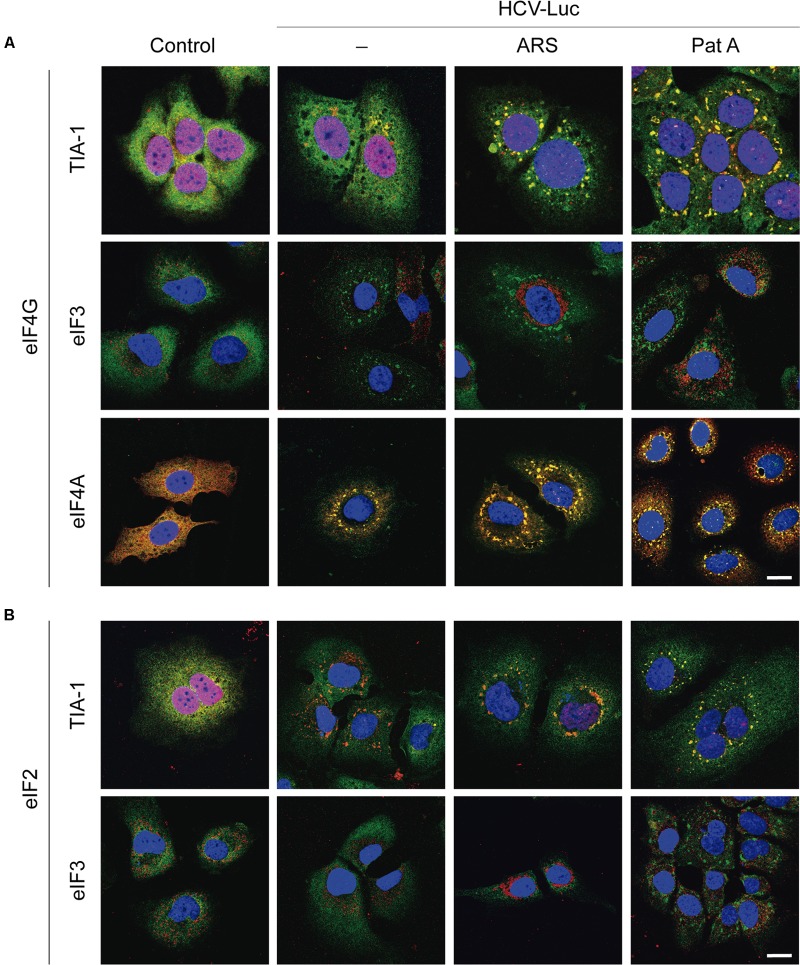
Analysis of stress granule formation in Huh-7 cells transfected with *in vitro* synthesized HCV-Luc RNA and treated with sodium arsenite or pateamine A. Cells were seeded on microscope cover slips, transfected with HCV-Luc mRNA for 1 h and then treated with either ARS (200 μM) or Pat A (0.4 μM) for 2 h. Control cells underwent the transfection procedure without RNA. After treatments, cells were permeabilized for immunocytochemistry. **(A)** Shows staining with primary rabbit anti-eIF4G antibody (green) together with primary goat anti-TIA-1, goat anti-eIF3 or mouse anti-eIF4A antibodies (red). **(B)** Shows staining with primary rabbit anti-eIF2 antibody (green) together with primary goat anti-TIA-1 or goat anti-eIF3 antibodies (red). Anti-goat antibodies conjugated to Alexa 555 were used to detect TIA-1 or eIF3 (red), anti-mouse antibody conjugated to Alexa 555 was used to detect eIF4A (red), anti-rabbit antibodies conjugated to Alexa 448 were employed to detect eIF4G or eIF2 (green). DAPI was used to stain the nuclei (blue). Scale bar, 20 μm.

To survey the action of ARS and Pat A on SG formation and to examine the localization of different eIFs, Huh-7 cells were transfected with HCV-Luc, incubated with ARS and Pat A for 2 h, and then processed for immunocytochemistry. We initially examined the localization of eIF4G together with TIA-1, eIF3 or eIF4A. As illustrated in **Figure 3A**, eIF4G (stained green) was mostly located in the cytoplasm in Huh-7 control cells, whereas TIA-1 (stained red) was predominantly nuclear. Transfection with HCV-Luc RNA led to the appearance of a few SGs in Huh-7 cells containing both eIF4G and TIA-1. Treatment of transfected cells with ARS or Pat A greatly increased the number and size of SGs containing TIA-1 and eIF4G (**Figure 3A**). eIF3 (stained red) commonly showed a granular and cytoplasmic location. Furthermore, transfection with HCV-Luc RNA led to the formation of some granules with concentrated eIF4G, presumably SGs, without co-localization of eIF3. However, ARS treatment robustly modified eIF3 distribution, which showed a more compacted perinuclear location without co-localization of eIF4G. This effect was not seen after Pat A treatment (**Figures 3A,B**). Moreover, whereas eIF4A (stained red) displayed a cytoplasmic location with a homogeneous dispersion in control cells, it was clearly present in small SGs coincident with eIF4G (stained green) in HCV-Luc transfected cells, which were larger after treatment with ARS or Pat A (**Figure 3A**). The finding that eIF4G is present in SGs suggests that it is not participating in the translation of HCV-Luc mRNA. This is also in agreement with the lack of inhibition when eIF4A is blocked by Hipp or Pat A, suggesting that the eIF4F complex is not involved in HCV mRNA translation. We also considered it of interest to analyze other factors of the translation machinery (**Figure 3B**). eIF2 (stained green) had a dispersed cytoplasmic localization in control and transfected cells with or without ARS treatment, whereas in transfected cells treated with Pat A eIF2 appeared in SGs coincident with TIA-1 (stained red) (**Figure 3B**). Curiously, Pat A not only blocked the action of eIF4A, but also induced the sequestration of eIF2 into SG granules. Finally, as shown in Supplementary Figure 2, no change in the localization pattern of eIF1A was found after RNA transfection with or without treatment with ARS or Pat A. Therefore, eIF1A is not sequestered into SGs. This is consistent with the idea that eIF1A may participate in the translation of HCV mRNA (Jaafar et al., 2016). In conclusion, eIF4G and eIF4A, which are located in SG, are not involved in HCV mRNA translation, whereas eIF3 and eIF1A, which are present in the cytosol, could be engage in the initiation of this translation. In the case of eIF2, it is located in SG after phosphorylation by Pat A, consistent with the concept that it does not participate in HCV IRES translation.

**FIGURE 4:**
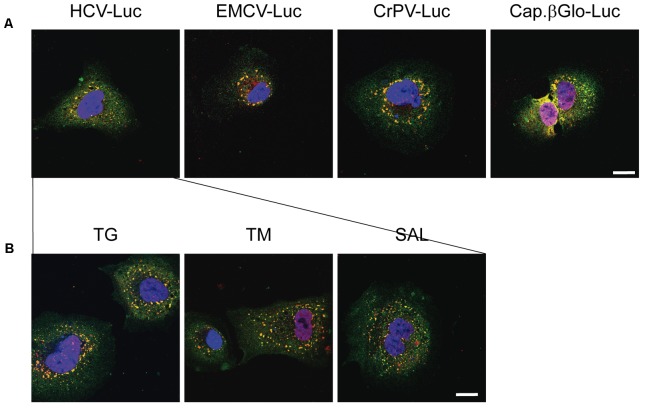
Thapsigargin, tunicamycin or salubrinal treatment induces stress granule formation in transfected Huh-7 cells. **(A)** Cells were seeded on microscope cover slips and transfected with different reporter RNAs: HCV-Luc, EMCV-Luc, CrPV-Luc or Cap.βGlobin-Luc. After 1 h of transfection, cells were incubated in DMEM medium for 2 h. **(B)** Cells were seeded on microscope cover slips and transfected with HCV-Luc. After 1 h of transfection, cells were treated with various eIF2 inhibitors (TG, TM, or SAL) for 2 h. In both cases, after treatments cells were permeabilized for immunocytochemistry using primary goat anti-TIA-1 and rabbit anti-eIF4G antibodies. An anti-goat antibody conjugated to Alexa 555 was used to detect TIA-1 (red) and an anti-rabbit antibody conjugated to Alexa 448 was employed to detect eIF4G (green). DAPI was used to stain the nuclei (blue). Scale bar, 20 μm.

We next addressed whether transfection with other RNAs could also induce the formation of SGs. We transfected Huh-7 cells with HCV-Luc (as a control), EMCV-Luc, CrPV-Luc or Cap.βGlobin-Luc RNAs for 1 h followed by incubation in DMEM for 2 h before immunocytochemistry analysis. Interestingly, the transfection with these RNAs also induced the formation of a few small granules (**Figure 4A**). This represent an interesting aspect to take into consideration, since not only HCV-Luc RNA but also transfection with other RNAs may induce this response. Of interest, treatment with TG, TM or SAL also increased the formation of large quantities of SGs in cells transfected with HCV-Luc mRNA as shown by staining for eIF4G (green) and TIA-1 (red) (**Figure 4B**).

### Translation of mRNA Bearing HCV IRES in Knock-out Human Cells Depleted for eIF2A, eIF2D, or Both Factors

Several studies have suggested that eIF2 can be replaced with other cellular proteins under stress conditions (Terenin et al., 2008; Dmitriev et al., 2010; Skabkin et al., 2010; Kim et al., 2011). Accordingly, the possibility that eIF2A or eIF2D participate in the initiation of translation of HCV mRNA when eIF2α is phosphorylated has been put forward. To test this idea, we made use of human cells knocked out for eIF2A (HAP1-eIF2A^-^), or eIF2D (HAP1-eIF2D^-^) or both (HAP1-eIF2A^-^/2D^-^). We have recently shown that these cells are viable, exhibit a normal morphology and have a similar synthesis of global proteins to that of parental control cells (Sanz et al., 2017).

**FIGURE 5:**
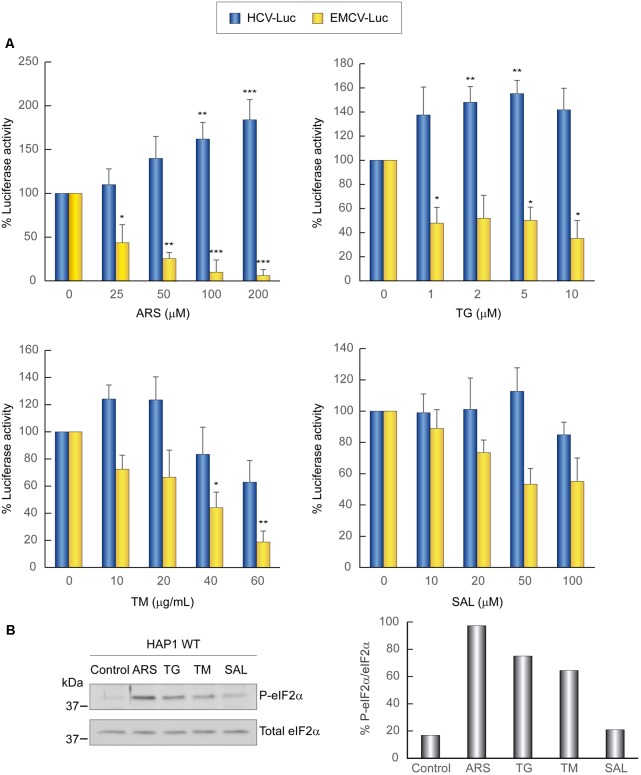
Translation from the HCV IRES in HAP1 WT cells is resistant to the action of eIF2 inhibitors. **(A)** Translation from the IRES of HCV or EMCV as measured by luciferase activity in response to eIF2 inhibitor treatment in HAP1 WT cells. Cells were transfected with HCV-Luc or EMCV-Luc mRNAs for 1 h and then incubated with either CHX (5 μg mL^-1^) or ARS, TG, TM, or SAL for a further 2 h. Percentage change is relative to non-treated control (inhibitor concentration = 0). The readings from CHX treatments were subtracted from all as a baseline. Error bars represent the standard error of the mean, *n* = 3. Statistical significance of the differences between treated samples compared to control was calculated with two-way ANOVA and a Bonferroni *post hoc* test, and is shown as: ^∗^*p* < 0.05, ^∗∗^*p* < 0.01, ^∗∗∗^*p* < 0.001. **(B)** Inhibitor treatment induces eIF2α phosphorylation in HAP1 WT cells. Cells were transfected for 1 h and then treated or not with either ARS (200 μM), TG (5 μM), TM (40 μg mL^-1^) or SAL (50 μM) for 2 h. Proteins were resolved using SDS-PAGE and then samples were probed with antibodies for phospho-eIF2α and total eIF2α. Shown is a representative blot from three independent experiments. The phosphorylation of eIF2α induction rate was evaluated by normalizing the raw value of P-eIF2α to that of total eIF2α as shown in the bar graph.

First, we analyzed the activity of the different inhibitors that induce the phosphorylation of eIF2α in WT HAP1 cells transfected with different mRNAs. We followed a protocol similar to that used for Huh-7 cells and each compound was assayed at different concentrations. Consistent with the results for hepatoma cells, the compounds differentially affected the translation directed by EMCV or HCV IRESs: whereas translation driven by EMCV IRES was inhibited, HCV IRES-dependent translation was stimulated by these compounds (**Figure 5A**). As stated earlier, this stimulation can be due to phosphorylation of eIF2α (**Figure 5B**), which blocks cellular translation, and also to the sequestration of many cellular mRNAs in SGs, impeding the competition for components of the translational machinery.

**FIGURE 6:**
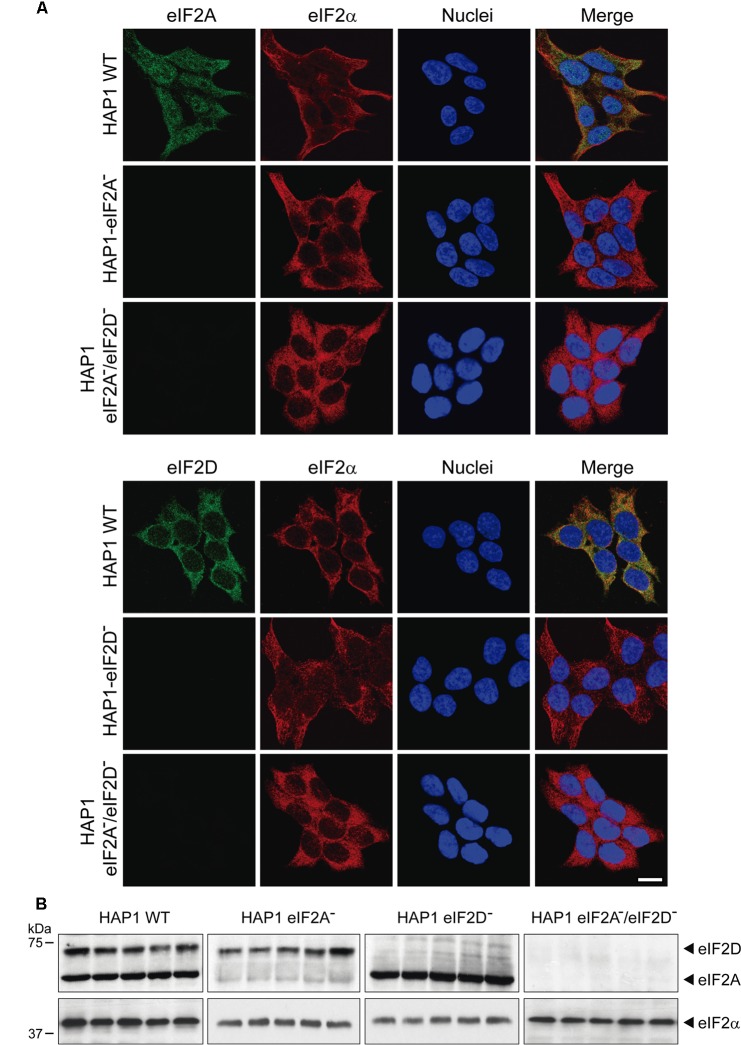
Characterization of the different HAP1 cell lines by immunocytochemistry and western blotting. **(A)** HAP1 WT, HAP1-eIF2A^-^, HAP1-eIF2D^-^, and HAP1-eIF2A^-^/eIF2D^-^ cells were seeded on microscope coverslips, fixed and stained with primary rabbit polyclonal anti-eIF2A or anti-eIF2D antibodies and a mouse monoclonal anti-eIF2α antibody. An anti-mouse antibody conjugated to Alexa 555 was used to detect eIF2α (red) and an anti-rabbit antibody conjugated to Alexa 488 was employed to detect eIF2A and eIF2D (green). DAPI was used to stain the nuclei (blue). Scale bar, 20 μm. **(B)** The presence of eIF2A or eIF2D in HAP1 WT, HAP1-eIF2A^-^, HAP1-eIF2D^-^, and HAP1-eIF2A^-^/eIF2D^-^ cells was also determined by western blotting with rabbit polyclonal anti-eIF2A and anti-eIF2D antibodies. eIF2α was used as loading control for the four cell lines. Proteins were resolved by SDS-PAGE and samples were probed with antibodies to show the presence of these factors in the HAP1 cells lines. Shown is a representative blot from three independent experiments.

To study the participation of eIF2A and eIF2D in the translation of the mRNA reporter bearing HCV IRES, we employed the KO cell lines indicated above. We first validated the cell lines by immunohistochemistry and western blotting. The subcellular localization of eIF2A and eIF2D in HAP1 WT, single KO and double KO variants is shown in **Figure 6**. The expression of eIF2A and eIF2D was examined by immunocytochemistry using specific antibodies. Double staining of HAP1 WT cells revealed that eIF2A was clearly expressed in the cytoplasm and a proportion was also found in the nucleus, whereas eIF2D was mainly cytoplasmic. As expected, eIF2A was not detected in HAP1-eIF2A^-^ cells or HAP1 eIF2A^-^/eIF2D^-^ (**Figure 6A**). Similarly, eIF2D was not found in HAP1-eIF2D^-^ cells or HAP1 eIF2A^-^/eIF2D^-^ (**Figure 6A**). Loss of eIF2A or eIF2D in the respective KO cell lines was verified by western blotting (**Figure 6B**).

**FIGURE 7:**
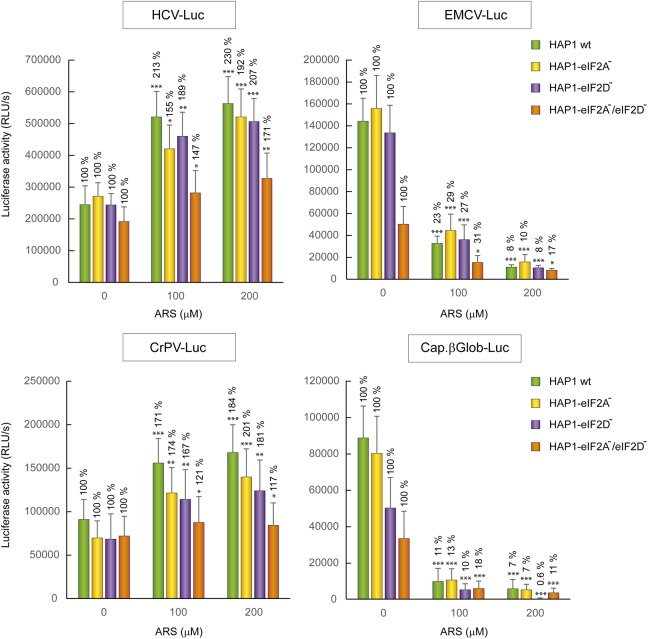
Resistance of HCV IRES translation to the eIF2 inhibitor sodium arsenite is independent of eIF2A and eIF2D. HAP1 cell lines WT, HAP1-eIF2A^-^, HAP1-eIF2D^-^, and HAP1-eIF2A^-^/eIF2D^-^ were transfected with reporter RNAs (HCV-Luc, EMCV-Luc, CrPV-Luc or Cap.βGlobin-Luc) for 1 h and treated with ARS (200 μM) for 2 h. Bars represent the relative luciferase activity with non-treated control (inhibitor concentration = 0) set as 100%. The readings from CHX treatments were subtracted from all as a baseline. Error bars represent the standard error of the mean, *n* = 3. Statistical significance of the differences between ARS treated samples compared to control was calculated with two-way ANOVA and a Bonferroni *post hoc* test, and is shown as: ^∗^*p* < 0.05, ^∗∗^*p* < 0.01, ^∗∗∗^*p* < 0.001.

The four cell lines, HAP1 WT, HAP1-eIF2A^-^, HAP1-eIF2D^-^, and HAP1-eIF2A^-^/2D^-^ were transfected with different mRNAs encoding luciferase and treated or not with ARS to induce eIF2α phosphorylation. Interestingly, the translation of the different mRNAs was similar in all four cell lines analyzed, both in the absence or presence of ARS (**Figure 7**). As expected, luciferase synthesis from both control mRNAs, EMCV-Luc and Cap.βGlo-Luc was strongly inhibited by ARS in all four cell lines. By contrast, luciferase translation from CrPV-Luc mRNA was stimulated by ARS treatment. Thus, HCV-Luc mRNA directed the synthesis of luciferase in all four cell lines, even when ARS was present and eIF2α was phosphorylated. This finding clearly demonstrates that neither eIF2A nor eIF2D are involved in HCV-Luc translation. Moreover, these two factors do not replace the activity of eIF2 in IRES-driven translation when it is inactivated. In the statistical analysis using two-way ANOVA, we have seen that there is no statistical interaction between any of the four HAP1 lines. That means that the effect of ARS is the same in the four cell lines, although there are differences between the levels of translation between them. The ARS increases in the same way the expression of Luc in the four lines tested despite the absence of eIF2A and/or eIF2D. Therefore the level of translation of HCV-Luc does not seem to depend on the presence of these factors. In turn, we also see that there is a significant difference between control cells and treated with ARS, as indicated in **Figure 7**, therefore ARS effectively stimulates the translation of HCV-Luc in the four HAP1 lines in a similar degree. These findings are consistent with a recent report showing that depleting Huh-7 cells of eIF2A, eIF2D or both with siRNAs has no effect on luciferase synthesis promoted by HCV IRES (Jaafar et al., 2016).

## Discussion

Animal viruses employ a variety of mechanisms to translate their mRNAs (Firth and Brierley, 2012). The precise mechanisms of viral mRNA translation remain the subject of intense research. Some animal viruses utilize RNA components known as IRES elements to direct the translation machinery to an internal position at the 5′-UTR of the viral messenger (Hellen, 2009; Plank and Kieft, 2012). However, the mechanism of IRES-driven translation also can differ between the various animal viruses that use this strategy. In the case of HCV, the exact mode by which its mRNA initiates protein synthesis as regards to participating eIFs is controversial. One school of thought is that under normal cellular conditions, HCV mRNA initiates translation using the ternary Met-tRNA_i_^Met^-eIF2-GTP complex, whereas eIF2 is dispensable under stress conditions (Jaafar et al., 2016). Yet, the possibility exists that eIF2 never participates in the initiation of HCV mRNA in infected cells. Indeed, the interaction of HCV IRES with the preinitiation complexes displaces eIF2 (Jaafar et al., 2016), and it is conceivable that the IRES element itself is sufficient to initiate translation without eIF2, even when this factor is active. Studies *in vitro* and experiments in culture cells indicate that eIF2A can replace eIF2 in HCV IRES-directed translation (Kim et al., 2011). Moreover, other studies point to eIF2D as the responsible factor to initiate translation in place of eIF2 (Dmitriev et al., 2010; Skabkin et al., 2010), based mainly on *in vitro* observations. Our present findings in human cells clearly demonstrate that neither eIF2A nor eIF2D are involved in protein synthesis directed by HCV IRES, and are in accord with a recent result demonstrating that knockdown of eIF2A or eIF2D in hepatoma cells has little effect on HCV IRES-driven translation (Jaafar et al., 2016). Therefore, the results obtained using siRNAs by Jaafar et al. and our present findings with KO cell lines are complementary and both demonstrate that those factors do not participate in HCV translation. In addition, the possibility that eIF2A can be replaced by eIF2D, or vice versa, is not supported by the finding that in the double KO cell line HCV-Luc mRNA is efficiently translated even under stress conditions in the presence of ARS. Moreover, elegant studies directed to uncover the cellular genes necessary for HCV replication and growth failed to detect eIF2A or eIF2D (Marceau et al., 2016). Although that study only examined cellular genes dispensable for human HAP1 cells, it must be stressed that, as we have demonstrated, neither eIF2A nor eIF2D are necessary for the viability of this cell line (Sanz et al., 2017). Therefore, we can conclude that these genes do not participate in the translation of this viral mRNA, and they do not replace eIF2 even after the induction of cellular stress. The use of KO cell lines will be helpful in future studies to unravel the mode of initiation and the factors required to translate viral mRNAs. Indeed, we believe that *in vitro* experiments that suggest the requirement for some eIFs to translate a given mRNA should be followed by *in vivo* experiments in culture cells and in this respect KO cells will be useful tools. Remarkably, all eIFs could be dispensable in *in vitro* translation of HCV at high concentrations of magnesium ions (Lancaster et al., 2006). Thus, the modulation of *in vitro* conditions strongly affects the requirements for eIFs in the initiation of protein synthesis by HCV IRES.

There is more consensus about the lack of any involvement of the three factors that form part of the eIF4F complex for the initiation of protein synthesis directed by HCV mRNA (Niepmann, 2013). Since this viral messenger does not have a 5′ cap structure, it seems logical that eIF4E is not involved in its translation. Also, since there is no scanning mechanism during the initiation event, eIF4A (the helicase enzyme involved in scanning) does not participate in this process. There are currently two compounds (Hipp and Pat A) from marine origin that are selective inhibitors of eIF4A (Bordeleau et al., 2006a; Low et al., 2007). Both are very useful to test the involvement of this factor in the translation of any given mRNA. We show here that these two agents do not affect luciferase synthesis driven by HCV IRES in monocistronic mRNAs containing the HCV 3′-UTR in human hepatic cells. Our results are in good agreement with previous observations showing that these compounds do not block HCV IRES-dependent translation in dicistronic mRNAs (Bordeleau et al., 2006a; Low et al., 2007). This fact, together with the finding that eIF4G are localized in SGs after ARS treatment, is consistent with the idea that the eIF4F complex does not participate in the initiation of translation of HCV mRNA.

Possibly the most important issue to be clarified in the initiation of protein synthesis driven by HCV IRES is to determine whether eIF2 is employed or not under normal conditions. *In vitro* observations have demonstrated that the HCV IRES can directly interact with native 40S ribosomal subunits devoid of eIFs that, afterward, can recruit eIF3 and the ternary complex containing eIF2 (Ji et al., 2004; Otto and Puglisi, 2004). The interaction of the HCV IRES with 40S or 80S ribosomes leads to the remodeling of its structure, in such a way that domain II is bound to the tRNA exit site, whereas domain III positions with the initiation codon at the head of the small ribosomal subunit (Boehringer et al., 2005). Fluorescently labeled 40S ribosomal subunits in the ribosomal protein RPS25 irreversibly bind to HCV IRES, leading to conformational rearrangements of domain II that are stabilized by yet undefined cellular proteins (Fuchs et al., 2015). It is, however, unclear if under physiological conditions in intact cells HCV mRNA interacts with native 40S or more probably with preinitiation complexes that would contain several eIFs, including eIF3 and eIF1A (Jaafar et al., 2016). IRES binding to preinitiation complexes in intact cells can displace eIF2 by the interaction of domain II with the 40S subunit (Locker et al., 2007). In fact, the binding sites of domain II and the ternary complex overlap in such a way that the interaction of both on the 40S would clash (Jaafar et al., 2016). It is feasible that domain II of HCV IRES functionally replaces the ternary complex, without the necessity for other cellular factors such as eIF2A or eIF2D even under normal cellular conditions. Indeed, domain II adopts an L-shaped structure and interacts with the 40S subunit in the head region of the E site, allowing the apical loop of domain II to reach deeply into the mRNA cleft near the coding RNA in the ribosomal P site (Spahn et al., 2001; Lukavsky et al., 2003). Therefore, the IRES may be able to replace the ternary complex and after the formation of the 80S ribosome, the P site could be occupied, leaving the A site free that could be positioned with the AUG initiation codon ready to start translation. This model is akin to that described for the functioning of CrPV IRES, with the exception that eIF3 is involved in HCV translation (Jan and Sarnow, 2002; Fernandez et al., 2014).

The exact functioning of eIF2A or eIF2D during cellular mRNA translation remains to be elucidated. The recent generation of an eIF2A knockout mouse clearly demonstrates that this factor is not required during embryogenesis, nor involved in the translation of tissue-specific mRNAs (Golovko et al., 2016). The recent finding that eIF2A participates in tumorigenesis makes the study of this factor particularly relevant (Sendoel et al., 2017). Nonetheless, eIF2A is likely required for additional functions besides its involvement in cancer progression. The use of the human KO cell lines employed in this work could help to improve our understanding of eIF2A and eIF2D in translation. As observed recently (Sanz et al., 2017), and in the present study, these two factors are not necessary for global translation of cellular mRNAs and for luciferase synthesis directed by HCV, EMCV, or CrPV IRESs. Future studies directed to analyze the behavior of specialized cellular mRNAs in these KO cell lines will help to ascertain the precise roles of eIF2A and eIF2D.

## Author Contributions

EG-A, HW, and MS performed the experiments. LC designed the experiments and wrote the manuscript. All authors listed have made a substantial, direct and intellectual contribution to the work, and approved it for publication.

## Conflict of Interest Statement

The authors declare that the research was conducted in the absence of any commercial or financial relationships that could be construed as a potential conflict of interest.

## References

[B1] Adams S. L. Safer B. Anderson W. F. Merrick W. C. (1975). Eukaryotic initiation complex formation. Evidence for two distinct pathways. *J. Biol. Chem.* 250 9083–9089. 1194278

[B2] Aldabe R. Carrasco L. (1995). Induction of membrane proliferation by poliovirus proteins 2C and 2BC. *Biochem. Biophys. Res. Commun.* 206 64–76. 10.1006/bbrc.1995.1010 7818552

[B3] Anderson P. Kedersha N. Ivanov P. (2015). Stress granules. P-bodies and cancer. *Biochim. Biophys. Acta* 1849 861–870. 10.1016/j.bbagrm.2014.11.009 25482014 PMC4457708

[B4] Bai Y. Zhou K. Doudna J. A. (2013). Hepatitis C virus 3′UTR regulates viral translation through direct interactions with the host translation machinery. *Nucleic Acids Res.* 41 7861–7874. 10.1093/nar/gkt543 23783572 PMC3763534

[B5] Boehringer D. Thermann R. Ostareck-Lederer A. Lewis J. D. Stark H. (2005). Structure of the hepatitis C virus IRES bound to the human 80S ribosome: remodeling of the HCV IRES. *Structure* 13 1695–1706. 10.1016/j.str.2005.08.008 16271893

[B6] Bordeleau M. E. Cencic R. Lindqvist L. Oberer M. Northcote P. Wagner G. (2006a). RNA-mediated sequestration of the RNA helicase eIF4A by pateamine A inhibits translation initiation. *Chem. Biol.* 13 1287–1295. 10.1016/j.chembiol.2006.10.005 17185224

[B7] Bordeleau M. E. Matthews J. Wojnar J. M. Lindqvist L. Novac O. Jankowsky E. (2005). Stimulation of mammalian translation initiation factor eIF4A activity by a small molecule inhibitor of eukaryotic translation. *Proc. Natl. Acad. Sci. U.S.A.* 102 10460–10465. 10.1073/pnas.0504249102 16030146 PMC1176247

[B8] Bordeleau M. E. Mori A. Oberer M. Lindqvist L. Chard L. S. Higa T. (2006b). Functional characterization of IRESes by an inhibitor of the RNA helicase eIF4A. *Nat. Chem. Biol.* 2 213–220. 10.1038/nchembio776 16532013

[B9] Boyce M. Bryant K. F. Jousse C. Long K. Harding H. P. Scheuner D. (2005). A selective inhibitor of eIF2alpha dephosphorylation protects cells from ER stress. *Science* 307 935–939. 10.1126/science.1101902 15705855

[B10] Castello A. Franco D. Moral-Lopez P. Berlanga J. J. Alvarez E. Wimmer E. (2009). HIV- 1 protease inhibits Cap- and poly(A)-dependent translation upon eIF4GI and PABP cleavage. *PLOS ONE* 4:e7997. 10.1371/journal.pone.0007997 19956697 PMC2776998

[B11] Dabo S. Meurs E. F. (2012). DsRNA-dependent protein kinase PKR and its role in stress, signaling and HCV infection. *Viruses* 4 2598–2635. 10.3390/v4112598 23202496 PMC3509664

[B12] Dang Y. Kedersha N. Low W. K. Romo D. Gorospe M. Kaufman R. (2006). Eukaryotic initiation factor 2alpha-independent pathway of stress granule induction by the natural product pateamine A. *J. Biol. Chem.* 281 32870–32878. 10.1074/jbc.M606149200 16951406

[B13] Dmitriev S. E. Terenin I. M. Andreev D. E. Ivanov P. A. Dunaevsky J. E. Merrick W. C. (2010). GTP-independent tRNA delivery to the ribosomal P-site by a novel eukaryotic translation factor. *J. Biol. Chem.* 285 26779–26787. 10.1074/jbc.M110.119693 20566627 PMC2930676

[B14] Fernandez I. S. Bai X. C. Murshudov G. Scheres S. H. Ramakrishnan V. (2014). Initiation of translation by cricket paralysis virus IRES requires its translocation in the ribosome. *Cell* 157 823–831. 10.1016/j.cell.2014.04.015 24792965 PMC4017093

[B15] Firth A. E. Brierley I. (2012). Non-canonical translation in RNA viruses. *J. Gen. Virol.* 93(Pt 7), 1385–1409. 10.1099/vir.0.042499-0 22535777 PMC3542737

[B16] Fuchs G. Petrov A. N. Marceau C. D. Popov L. M. Chen J. O’Leary S. E. (2015). Kinetic pathway of 40S ribosomal subunit recruitment to hepatitis C virus internal ribosome entry site. *Proc. Natl. Acad. Sci. U.S.A.* 112 319–325. 10.1073/pnas.1421328111 25516984 PMC4299178

[B17] Garcia-Moreno M. Sanz M. A. Carrasco L. (2015). Initiation codon selection is accomplished by a scanning mechanism without crucial initiation factors in Sindbis virus subgenomic mRNA. *RNA* 21 93–112. 10.1261/rna.047084.114 25404563 PMC4274640

[B18] Garcia-Moreno M. Sanz M. A. Pelletier J. Carrasco L. (2013). Requirements for eIF4A and eIF2 during translation of Sindbis virus subgenomic mRNA in vertebrate and invertebrate host cells. *Cell Microbiol.* 15 823–840. 10.1111/cmi.12079 23189929

[B19] Golovko A. Kojukhov A. Guan B. J. Morpurgo B. Merrick W. C. Mazumder B. (2016). The eIF2A knockout mouse. *Cell Cycle* 15 k3115–3120. 10.1080/15384101.2016.1237324 27686860 PMC5134716

[B20] Gonzalez-Almela E. Sanz M. A. Garcia-Moreno M. Northcote P. Pelletier J. Carrasco L. (2015). Differential action of pateamine A on translation of genomic and subgenomic mRNAs from Sindbis virus. *Virology* 484 41–50. 10.1016/j.virol.2015.05.002 26057151

[B21] Hajarizadeh B. Grebely J. Dore G. J. (2013). Epidemiology and natural history of HCV infection. *Nat. Rev. Gastroenterol. Hepatol.* 10 553–562. 10.1038/nrgastro.2013.107 23817321

[B22] Hellen C. U. (2009). IRES-induced conformational changes in the ribosome and the mechanism of translation initiation by internal ribosomal entry. *Biochim. Biophys. Acta* 1789 558–570. 10.1016/j.bbagrm.2009.06.001 19539793 PMC2935204

[B23] Hellen C. U. Pestova T. V. (1999). Translation of hepatitis C virus RNA. *J. Viral. Hepat.* 6 79–87. 10.1046/j.1365-2893.1999.00150.x 10607219

[B24] Hinnebusch A. G. Ivanov I. P. Sonenberg N. (2016). Translational control by 5′-untranslated regions of eukaryotic mRNAs. *Science* 352 1413–1416. 10.1126/science.aad9868 27313038 PMC7422601

[B25] Ito T. Tahara S. M. Lai M. M. (1998). The 3′-untranslated region of hepatitis C virus RNA enhances translation from an internal ribosomal entry site. *J. Virol.* 72 8789–8796. 9765423 10.1128/jvi.72.11.8789-8796.1998 PMC110295

[B26] Jaafar Z. A. Oguro A. Nakamura Y. Kieft J. S. (2016). Translation initiation by the hepatitis C virus IRES requires eIF1A and ribosomal complex remodeling. *Elife* 5:e21198. 10.7554/eLife.21198 28009256 PMC5238962

[B27] Jan E. Sarnow P. (2002). Factorless ribosome assembly on the internal ribosome entry site of cricket paralysis virus. *J. Mol. Biol.* 324 889–902. 10.1016/S0022-2836(02)01099-9 12470947

[B28] Ji H. Fraser C. S. Yu Y. Leary J. Doudna J. A. (2004). Coordinated assembly of human translation initiation complexes by the hepatitis C virus internal ribosome entry site RNA. *Proc. Natl. Acad. Sci. U.S.A.* 101 16990–16995. 10.1073/pnas.0407402101 15563596 PMC534415

[B29] Kedersha N. Anderson P. (2009). Regulation of translation by stress granules and processing bodies. *Prog. Mol. Biol. Transl. Sci.* 90 155–185. 10.1016/S1877-1173(09)90004-7 20374741 PMC7102815

[B30] Khawaja A. Vopalensky V. Pospisek M. (2015). Understanding the potential of hepatitis C virus internal ribosome entry site domains to modulate translation initiation via their structure and function. *Wiley Interdiscip. Rev. RNA* 6 211–224. 10.1002/wrna.1268 25352252 PMC4361049

[B31] Khullar V. Firpi R. J. (2015). Hepatitis C cirrhosis: new perspectives for diagnosis and treatment. *World J. Hepatol.* 7 1843–1855. 10.4254/wjh.v7.i14.1843 26207166 PMC4506942

[B32] Kim J. H. Park S. M. Park J. H. Keum S. J. Jang S. K. (2011). EIF2A mediates translation of hepatitis C viral mRNA under stress conditions. *EMBO J.* 30 2454–2464. 10.1038/emboj.2011.146 21556050 PMC3116280

[B33] Lancaster A. M. Jan E. Sarnow P. (2006). Initiation factor-independent translation mediated by the hepatitis C virus internal ribosome entry site. *RNA* 12 894–902. 10.1261/rna.2342306 16556939 PMC1440913

[B34] Liang H. He S. Yang J. Jia X. Wang P. Chen X. (2014). PTENalpha, a PTEN isoform translated through alternative initiation, regulates mitochondrial function and energy metabolism. *Cell Metab.* 19 836–848. 10.1016/j.cmet.2014.03.023 24768297 PMC4097321

[B35] Linero F. N. Thomas M. G. Boccaccio G. L. Scolaro L. A. (2011). Junin virus infection impairs stress-granule formation in Vero cells treated with arsenite via inhibition of eIF2alpha phosphorylation. *J. Gen. Virol.* 92(Pt 12), 2889–2899. 10.1099/vir.0.033407-0 21813702

[B36] Locker N. Easton L. E. Lukavsky P. J. (2007). HCV and CSFV IRES domain II mediate eIF2 release during 80S ribosome assembly. *EMBO J.* 26 795–805. 10.1038/sj.emboj.7601549 17255934 PMC1794401

[B37] Low W. K. Dang Y. Schneider-Poetsch T. Shi Z. Choi N. S. Rzasa R. M. (2007). Isolation and identification of eukaryotic initiation factor 4A as a molecular target for the marine natural product Pateamine A. *Methods Enzymol.* 431 303–324. 10.1016/S0076-6879(07)31014-8 17923240

[B38] Lukavsky P. J. (2009). Structure and function of HCV IRES domains. *Virus Res.* 139 166–171. 10.1016/j.virusres.2008.06.004 18638512 PMC2726286

[B39] Lukavsky P. J. Kim I. Otto G. A. Puglisi J. D. (2003). Structure of HCV IRES domain II determined by NMR. *Nat. Struct. Biol.* 10 1033–1038. 10.1038/nsb1004 14578934

[B40] Madan V. Castello A. Carrasco L. (2008). Viroporins from RNA viruses induce caspase-dependent apoptosis. *Cell. Microbiol.* 10 437–451. 10.1111/j.1462-5822.2007.01057.x 17961183 PMC7162385

[B41] Marceau C. D. Puschnik A. S. Majzoub K. Ooi Y. S. Brewer S. M. Fuchs G. (2016). Genetic dissection of Flaviviridae host factors through genome-scale CRISPR screens. *Nature* 535 159–163. 10.1038/nature18631 27383987 PMC4964798

[B42] Merrick W. C. Anderson W. F. (1975). Purification and characterization of homogeneous protein synthesis initiation factor M1 from rabbit reticulocytes. *J. Biol. Chem.* 250 1197–1206. 1112800

[B43] Moss B. Elroy-Stein O. Mizukami T. Alexander W. A. Fuerst T. R. (1990). Product review. New mammalian expression vectors. *Nature* 348 91–92. 10.1038/348091a0 2234068

[B44] Nakabayashi H. Taketa K. Miyano K. Yamane T. Sato J. (1982). Growth of human hepatoma cells lines with differentiated functions in chemically defined medium. *Cancer Res.* 42 3858–3863. 6286115

[B45] Niepmann M. (2013). Hepatitis C virus RNA translation. *Curr. Top. Microbiol. Immunol.* 369 143–166. 10.1007/978-3-642-27340-7_6 23463200

[B46] Otto G. A. Puglisi J. D. (2004). The pathway of HCV IRES-mediated translation initiation. *Cell* 119 369–380. 10.1016/j.cell.2004.09.038 15507208

[B47] Paul D. Madan V. Bartenschlager R. (2014). Hepatitis C virus RNA replication and assembly: living on the fat of the land. *Cell Host Microbe* 16 569–579. 10.1016/j.chom.2014.10.008 25525790 PMC7172941

[B48] Penas C. Mascarenas J. L. Vazquez M. E. (2016). Coupling the folding of a beta-hairpin with chelation-enhanced luminescence of Tb(III) and Eu(III) ions for specific sensing of a viral RNA. *Chem. Sci.* 2016 2674–2678. 10.1039/C5SC04501K 27293537 PMC4898589

[B49] Pestova T. V. Shatsky I. N. Fletcher S. P. Jackson R. J. Hellen C. U. (1998). A prokaryotic-like mode of cytoplasmic eukaryotic ribosome binding to the initiation codon during internal translation initiation of hepatitis C and classical swine fever virus RNAs. *Genes Dev.* 12 67–83. 10.1101/gad.12.1.67 9420332 PMC316404

[B50] Plank T. D. Kieft J. S. (2012). The structures of nonprotein-coding RNAs that drive internal ribosome entry site function. *Wiley Interdiscip. Rev. RNA* 3 195–212. 10.1002/wrna.1105 22215521 PMC3973487

[B51] Redondo N. Sanz M. A. Welnowska E. Carrasco L. (2011). Translation without eIF2 promoted by poliovirus 2A protease. *PLOS ONE* 6:e25699. 10.1371/journal.pone.0025699 22003403 PMC3189197

[B52] Reynolds J. E. Kaminski A. Kettinen H. J. Grace K. Clarke B. E. Carroll A. R. (1995). Unique features of internal initiation of hepatitis C virus RNA translation. *EMBO J.* 14 6010–6020. 8846793 10.1002/j.1460-2075.1995.tb00289.x PMC394721

[B53] Robert F. Kapp L. D. Khan S. N. Acker M. G. Kolitz S. Kazemi S. (2006). Initiation of protein synthesis by hepatitis C virus is refractory to reduced eIF2.GTP.Met-tRNA(i)(Met) ternary complex availability. *Mol. Biol. Cell* 17 4632–4644. 10.1091/mbc.E06-06-0478 16928960 PMC1635388

[B54] Ruggieri A. Dazert E. Metz P. Hofmann S. Bergeest J. P. Mazur J. (2012). Dynamic oscillation of translation and stress granule formation mark the cellular response to virus infection. *Cell Host Microbe* 12 71–85. 10.1016/j.chom.2012.05.013 22817989 PMC3873964

[B55] Sanz M. A. Castello A. Ventoso I. Berlanga J. J. Carrasco L. (2009). Dual mechanism for the translation of subgenomic mRNA from Sindbis virus in infected and uninfected cells. *PLOS ONE* 4:e4772. 10.1371/journal.pone.0004772 19274090 PMC2651626

[B56] Sanz M. A. Gonzalez Almela E. Carrasco L. (2017). Translation of Sindbis subgenomic mRNA is independent of eIF2, eIF2A and eIF2D. *Sci. Rep.* 7:43876. 10.1038/srep43876 28240315 PMC5327398

[B57] Sanz M. A. Welnowska E. Redondo N. Carrasco L. (2010). Translation driven by picornavirus IRES is hampered from Sindbis virus replicons: rescue by poliovirus 2A protease. *J. Mol. Biol.* 402 101–117. 10.1016/j.jmb.2010.07.014 20643140

[B58] Schaller T. Appel N. Koutsoudakis G. Kallis S. Lohmann V. Pietschmann T. (2007). Analysis of hepatitis C virus superinfection exclusion by using novel fluorochrome gene-tagged viral genomes. *J. Virol.* 81 4591–4603. 10.1128/JVI.02144-06 17301154 PMC1900174

[B59] Sendoel A. Dunn J. G. Rodriguez E. H. Naik S. Gomez N. C. Hurwitz B. (2017). Translation from unconventional 5′ start sites drives tumour initiation. *Nature* 541 494–499. 10.1038/nature21036 28077873 PMC5287289

[B60] Shimoike T. McKenna S. A. Lindhout D. A. Puglisi J. D. (2009). Translational insensitivity to potent activation of PKR by HCV IRES RNA. *Antiviral Res.* 83 228–237. 10.1016/j.antiviral.2009.05.004 19467267

[B61] Shwetha S. Kumar A. Mullick R. Vasudevan D. Mukherjee N. Das S. (2015). HuR displaces polypyrimidine tract binding protein to facilitate la binding to the 3′ untranslated region and enhances hepatitis C virus replication. *J. Virol.* 89 11356–11371. 10.1128/JVI.01714-15 26339049 PMC4645635

[B62] Skabkin M. A. Skabkina O. V. Dhote V. Komar A. A. Hellen C. U. Pestova T. V. (2010). Activities of Ligatin and MCT-1/DENR in eukaryotic translation initiation and ribosomal recycling. *Genes Dev.* 24 1787–1801. 10.1101/gad.1957510 20713520 PMC2922506

[B63] Sonenberg N. Hinnebusch A. G. (2009). Regulation of translation initiation in eukaryotes: mechanisms and biological targets. *Cell* 136 731–745. 10.1016/j.cell.2009.01.042 19239892 PMC3610329

[B64] Spahn C. M. Kieft J. S. Grassucci R. A. Penczek P. A. Zhou K. Doudna J. A. (2001). Hepatitis C virus IRES RNA-induced changes in the conformation of the 40s ribosomal subunit. *Science* 291 1959–1962. 10.1126/science.1058409 11239155

[B65] Starck S. R. Tsai J. C. Chen K. Shodiya M. Wang L. Yahiro K. (2016). Translation from the 5’ untranslated region shapes the integrated stress response. *Science* 351:aad3867. 10.1126/science.aad3867 26823435 PMC4882168

[B66] Terenin I. M. Dmitriev S. E. Andreev D. E. Shatsky I. N. (2008). Eukaryotic translation initiation machinery can operate in a bacterial-like mode without eIF2. *Nat. Struct. Mol. Biol.* 15 836–841. 10.1038/nsmb.1445 18604219

[B67] Topisirovic I. Svitkin Y. V. Sonenberg N. Shatkin A. J. (2011). Cap and cap-binding proteins in the control of gene expression. *Wiley Interdiscip. Rev. RNA* 2 277–298. 10.1002/wrna.52 21957010

[B68] Valadao A. L. Aguiar R. S. de Arruda L. B. (2016). Interplay between inflammation and cellular stress triggered by flaviviridae viruses. *Front. Microbiol.* 7:1233. 10.3389/fmicb.2016.01233 27610098 PMC4996823

[B69] Vaughn L. S. Snee B. Patel R. C. (2014). Inhibition of PKR protects against tunicamycin-induced apoptosis in neuroblastoma cells. *Gene* 536 90–96. 10.1016/j.gene.2013.11.074 24334130

[B70] Welnowska E. Sanz M. A. Redondo N. Carrasco L. (2011). Translation of viral mRNA without active eIF2: the case of picornaviruses. *PLOS ONE* 6:e22230. 10.1371/journal.pone.0022230 21779397 PMC3136507

[B71] Yamamoto H. Collier M. Loerke J. Ismer J. Schmidt A. Hilal T. (2015). Molecular architecture of the ribosome-bound hepatitis C virus internal ribosomal entry site RNA. *EMBO J.* 34 3042–3058. 10.15252/embj.201592469 26604301 PMC4687786

[B72] Zoll W. L. Horton L. E. Komar A. A. Hensold J. O. Merrick W. C. (2002). Characterization of mammalian eIF2A and identification of the yeast homolog. *J. Biol. Chem.* 277 37079–37087. 10.1074/jbc.M207109200 12133843

