# Supplementary material for: The Initiation Factors eIF2, eIF2A, eIF2D, eIF4A, and eIF4G Are Not Involved in Translation Driven by Hepatitis C Virus IRES in Human Cells
Source: Front Microbiol. 2018 Feb 13;9:207. doi: 10.3389/fmicb.2018.00207 (PMC5816946; doi:10.3389/fmicb.2018.00207)
Supplement: Supplementary file 1 [file Data_Sheet_1.DOCX]

The initiation factors eIF2, eIF2A, eIF2D, eIF4A and eIF4G are not involved in translation driven by Hepatitis C virus IRES in human cells

Esther González Almela, Hugh Williams, Miguel Angel Sanz and Luis Carrasco^*^

Centro de Biología Molecular Severo Ochoa (CSIC-UAM). c/Nicolás Cabrera, 1. Universidad Autónoma de Madrid. 28049 Madrid. Spain

^*^Correspondence: +34-91-1964517

Running title: Translation of hepatitis C virus RNA

Key words: Regulation of protein synthesis, initiation factor of translation, inhibitors of eIF2, regulation of viral translation, eIF2 phosphorylation

**Supplementary Figure 1.** Schematic representation of the constructs used as templates for *in vitro* transcription of the mRNAs employed.


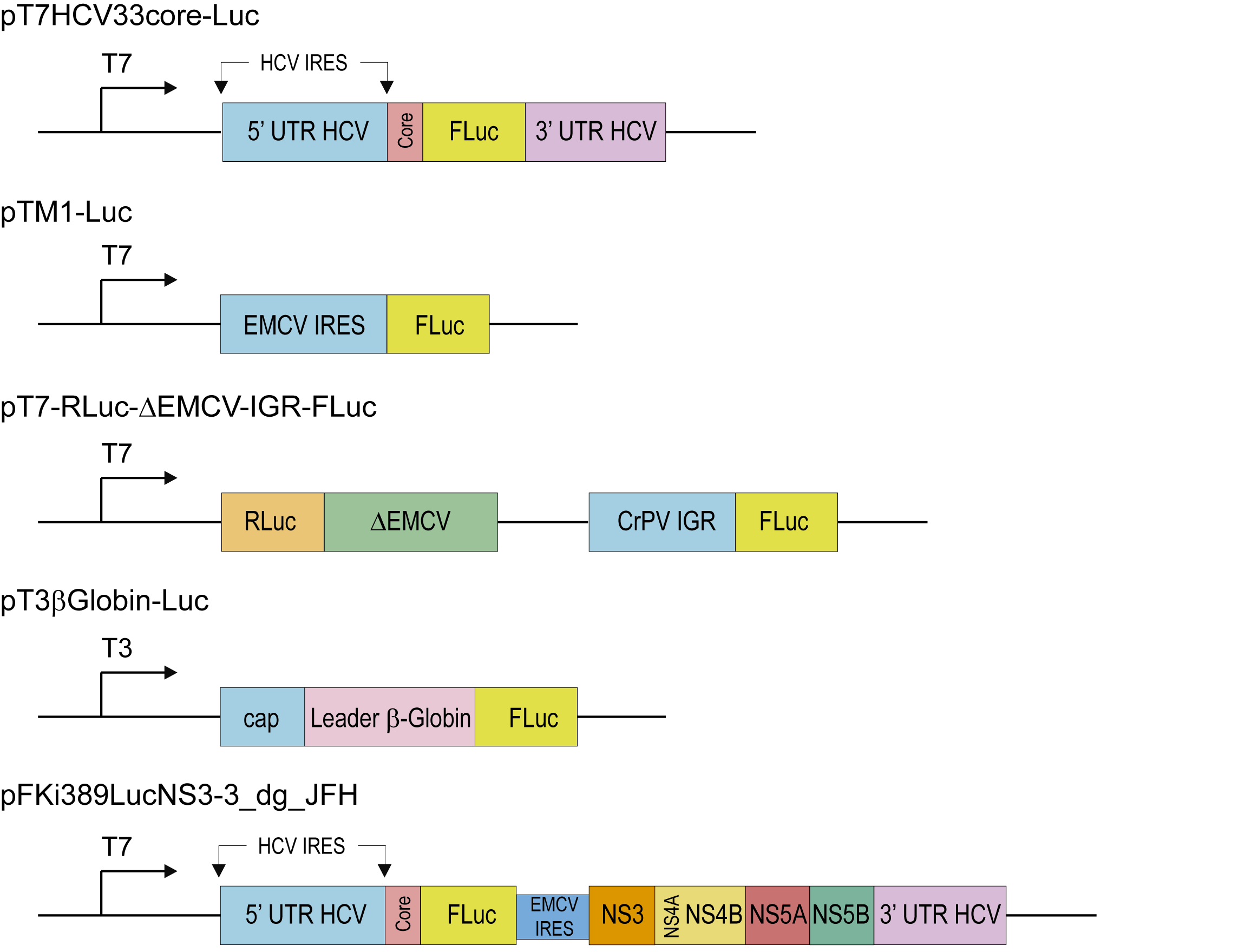

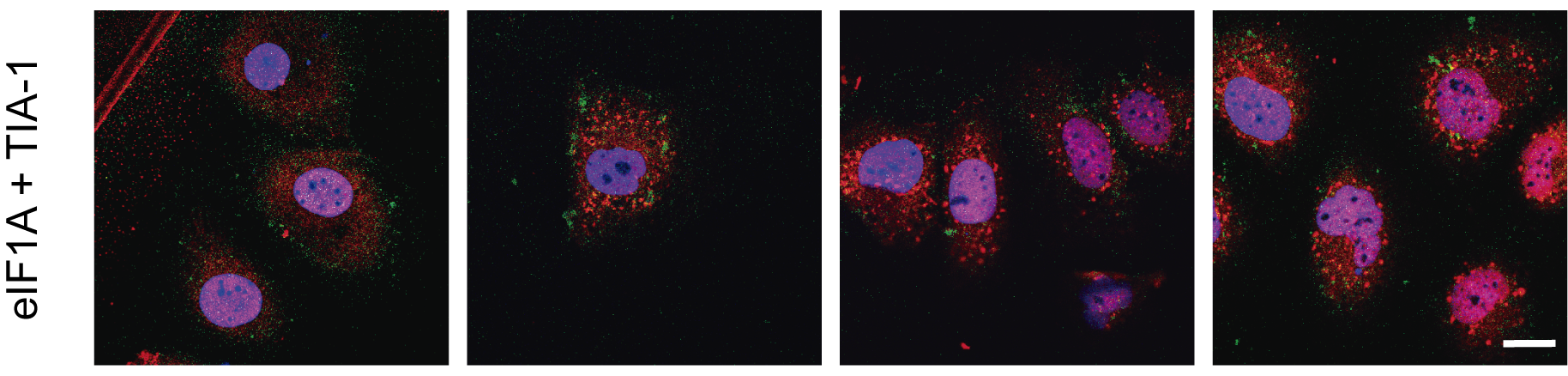


**Supplementary Figure 2. Analysis of eIF1A location and stress granule formation.**

Cells were seeded on microscope cover slips, transfected with HCV-Luc mRNA for 1 h and then treated with either ARS (200 μM) or Pat A (0.4 μM) for 2 h. Control cells underwent the transfection procedure without RNA. After treatment, cells were permeabilized for immunocytochemistry. Primary rabbit anti-eIF1A antibody (green) together with primary goat anti-TIA-1 antibody (red) were used. An anti-goat antibody conjugated to Alexa 555 was used to detect TIA-1 (red), and an anti-rabbit antibody conjugated to Alexa 448 was employed to detect eIF1A (green). DAPI was used to stain the nuclei (blue). Scale bar, 20 μm.
